# The complete annotated mitochondrial genome of *Cerastoderma glaucum* (Bruguière, 1789) from the Baltic Sea

**DOI:** 10.1080/23802359.2025.2584950

**Published:** 2025-11-11

**Authors:** Beata Śmietanka

**Affiliations:** Department of Genetics and Marine Biotechnology, Institute of Oceanology Polish Academy of Sciences, Sopot, Poland

**Keywords:** Bivalve, North Atlantic, mitogenomics, NGS, Cardiidae

## Abstract

*Cerastoderma glaucum is a key* intertidal bivalve in the north-east Atlantic*,* serving as a trophic resource for fish and birds. Its mitochondrial genome comprises 14,953 bp, encoding 13 protein-coding genes, 2 rRNA genes, and 22 tRNA genes. Gene structure resembles *Cerastoderma edule* but lacks an additional methionine tRNA. Phylogenetic analysis revealed close affinity between Baltic and English samples (p-distance = 0.027). No mitochondrial heteroplasmy was detected in mature male gonads, suggesting *C. glaucum* is not a doubly uniparental inheritance (DUI) species. These findings contribute to understanding mitogenomic variation and reproductive biology in bivalves inhabiting dynamic coastal ecosystems .

## Introduction

Cockles are considered a very important component of the coastal marine ecosystem and a major food source for many species, especially fish and birds exploring the intertidal environment. Euryhaline bivalve mollusc *C. glaucum* (Bruguière, 1789) is one of the two main cockles occurring in the coastal areas of the north-east Atlantic from Norway to Senegal, through the Baltic, Mediterranean, Black Sea, the Caspian Sea, and even the Aral Lake, in salinities from 4 to 100 ppt (Russell and Petersen 1973; Hayward and Ryland 1995; Nikula and Väinölä 2003; Malham et al. 2012). *C. glaucum* prefers non-tidal areas such as lagoons or salt marshes unlike the exploited edible *C. edule* often found on open coasts and estuaries (Reise 2003). The patterns of inter as well as intra-species genetic diversity are essential in the context of exact identification, management, and protection of natural resources. Cockles are considered important ecosystem engineers that contribute to a range of ecological functions (Carss et al. 2020). The reported declines of cockle populations noticed for many years due to climate events (Rowley et al. 2014), mass mortalities produced by pollution, predation, disease, parasite (De Montaudouin et al. 2009; Longshaw and Malham 2013), failed recruitment and probably most importantly over-fishing (Ducrotoy et al. 1989; Burdon et al. 2014; Villalba et al. 2014) underscores and justifies the critical importance of expanding the genetic resource base for these species (Mahony et al. 2020; Pampín et al. 2023).

Mitochondrial DNA is a useful marker for species identification, phylogeographic and phylogenetic analyses. However, in many bivalve species, a unique doubly uniparental inheritance (DUI) of mtDNA is observed (Skibinski et al. 1994; Zouros et al. 1994). Under this model, heteroplasmic males possess an additional divergent male mitogenome located in gametes and transmitted exclusively to the sons, homoplasmic females possess only female mtDNA transmitted to both offspring. The existence of such a marker seriously complicates the interpretation of mitochondrial DNA-based research; hence, it is crucial to positively identify DUI species.

The complete mitogenome of *C. edule* is available in GenBank (MF374632) but for the second cockle species *C. glaucum*, there is published online non-annotated sequence (OZ205200) from England. It is also unclear if these cockles are DUI species.

Here, we report the analysis of the next generation sequencing (NGS) data coming from mature male gonads of *C. glaucum* sampled in the Baltic Sea, filling this gap.

## Materials and methods

**Figure 1.**
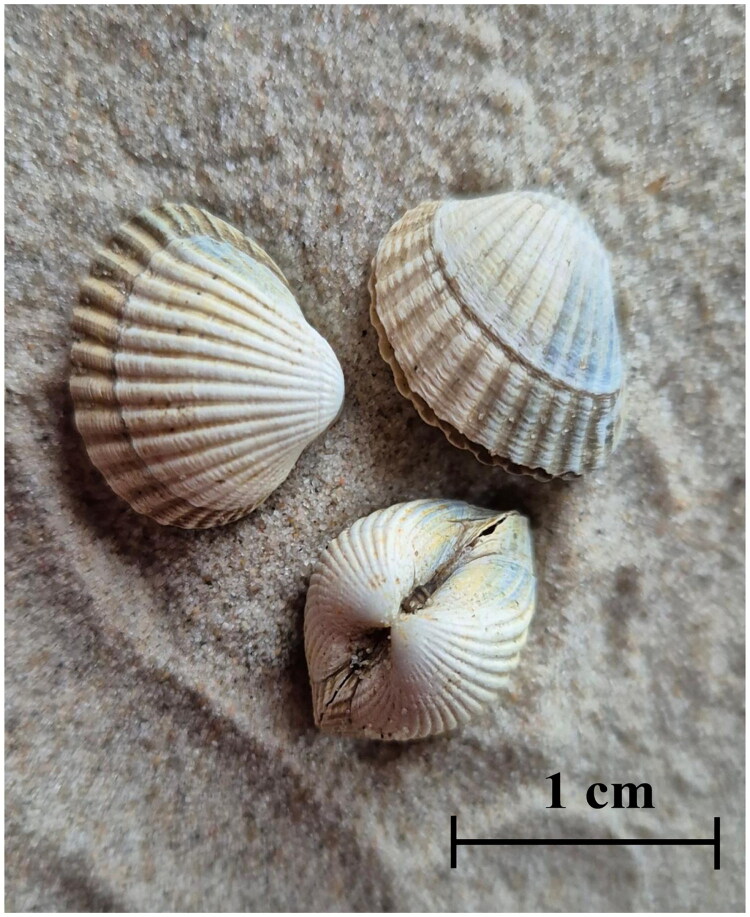
Image of the species of *Cerastoderma glaucum* from the Baltic Sea. The photo was taken by Beata Śmietanka.

The sample of adult *C. glaucum* (Figure 1) was collected in July 2020 from the Baltic Sea (54.668 N 18.673 E) as the only cockle of *Cerastoderma* in this sea (Piechocki and Wawrzyniak-Wydrowska 2016). Cockles were sexed by gonads analysis under the light microscope (presence of sperm cells or eggs) and stored at −70 °C. To verify the potential transmission of mtDNA to the offspring according to the DUI, the male specimen was selected for further analysis. Preserved gonadal tissue was deposited at the Institute of Oceanology PAN, Sopot, Poland; contact person and email: Beata Śmietanka bsmietanka@iopan.pl under the voucher number CG072020. Total DNA extraction from gonads was performed according to the CTAB method (Hoarau et al. 2002). DNA was quality checked by spectrophotometric assessment of concentration, then sent to Macrogen Inc. (Seoul, South Korea) for NGS approach. They prepared the TruSeq NGS library and sequenced it on the Illumina NovaSeq 6000 platform (San Diego, CA), yielding 45,484,508 raw, PE, 150 bp long reads.

A preliminary assembly of raw reads was performed in MEGAHIT (Li et al. 2015), with a k-mer size of 100 bp. The obtained contigs were searched for mitochondrial protein signatures by Wise2 (Birney et al. 2004) and each of them was used as a bait in NOVOPlasty (Dierckxsens et al. 2017). Each NOVOPlasty assembly yielded the same, circularized mitochondrial contig, which was validated by mapping raw reads back on the assembly to the mitogenomes in CLC Genomics Workbench 9.5.5 (QIAGEN, Hilden, Germany). The depth of coverage obtained by mapping raw sequencing reads onto the *C. glaucum* mitochondrial genome assembly is presented in Supplementary Figure S1. For gene prediction and annotation, the MITOCONSTRICTOR (Lubośny et al. 2022) python script with default parameters running and combining results from the following bioinformatic tools: CRITICA (Badger and Olsen 1999), Wise2 (Birney et al. 2004), GLIMMER (Delcher et al. 1999), ARWEN (Laslett and Canbäck 2008), and Infernal (Nawrocki and Eddy 2013) was used.

For comparative analysis, 11 Cardiidae family mitogenomes were selected based on finding regions of similarity between sequences in Nucleotide BLAST+ (Camacho et al. 2009). The pairwise genetic distance between mitogenomes was calculated as nucleotide *p*-distance in MEGA11 (Tamura et al. 2021). Reconstructions of phylogenetic relationships, based on 13 protein-coding and two rRNA genes alignment were performed using two programs: BEAST2 (Bouckaert et al. 2014) and Iqtree2 (Minh et al. 2020).

## Results

**Figure 2.**
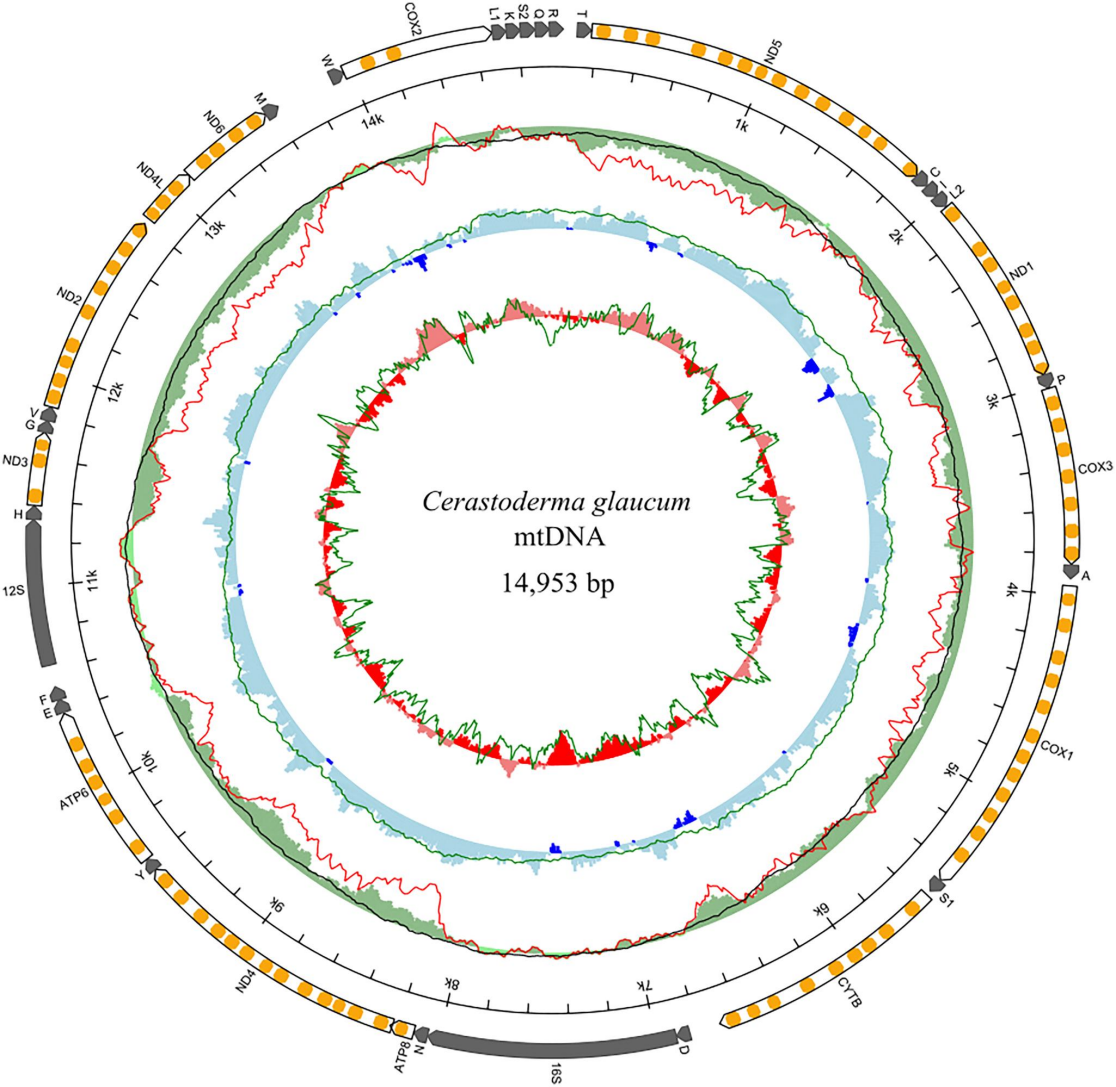
Genetic map of *Cerastoderma glaucum* mitochondrial genome. The white arrows with orange bands represent 13 protein-coding genes with predicted transmembrane domains. The dark arrows represent two rRNA and 22 tRNA genes. The green outer circle reflects the AT-skew and the red line on this circle represents filtered AT-skew, calculated at non-coding regions and the second codon position. A black line represents a filtered AT-skew, calculated at neutral and non-coding positions only. The middle blue circle presenting GC skew is connected with the green line for GC skew at neutral positions. The inner red circle shows local GC content, and the green line reflects GC content at neutral sites. The map and compositional indices were generated with MITOCONSTRICTOR (https://github.com/aburzynski/mitoconstrictor). Transmembrane domains in amino acid sequences of the proteins were predicted with TMHMM (Krogh et al. 2001).

**Figure 3.**
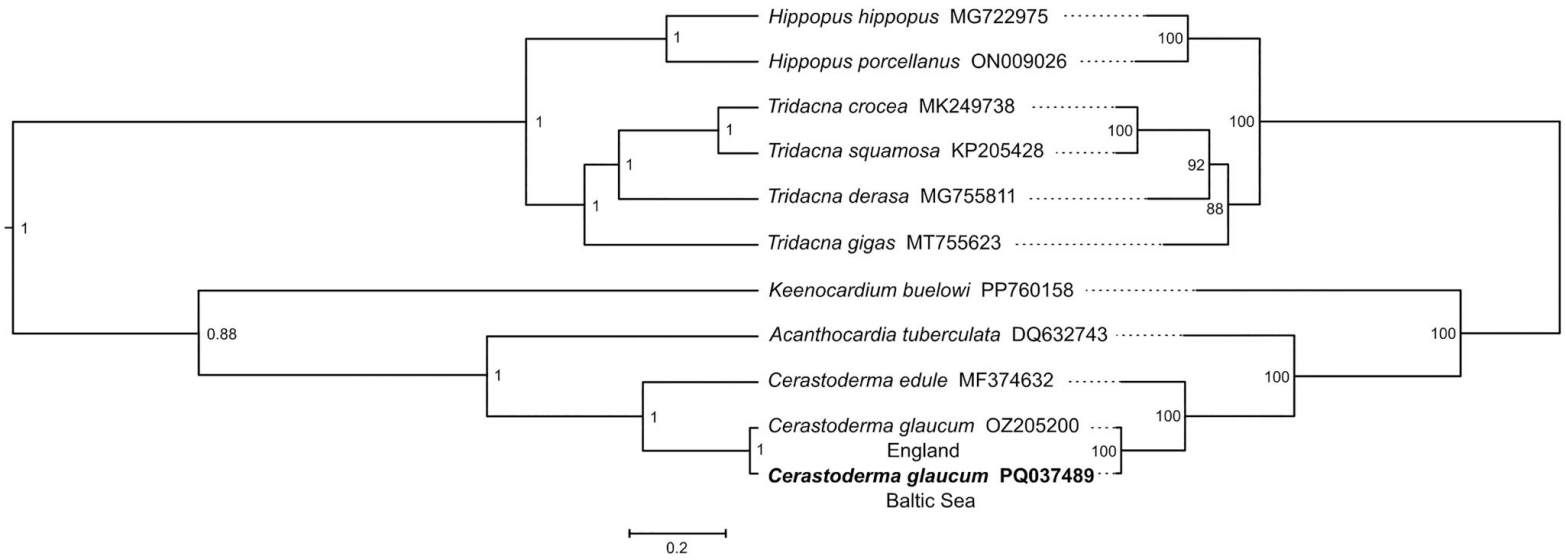
Phylogenetic tree based on comparative analysis of the announced mitogenome of C*erastoderma glaucum* from the Baltic Sea (in bold) and 10 mitogenome sequences chosen by Nucleotide BLAST+: *Acanthocardia tuberculata* (DQ632743, Dreyer and Steiner 2006), *Cerastoderma edule* (MF374632, unpublished), *Cerastoderma glaucum* (OZ205200, Wellcome Sanger Tree of Life Programme 2024), *Hippopus hippopus* (MG722975, Ma et al. 2019), *Hippopus porcellanus* (ON009026, Ma et al. 2022), *Keenocardium buelowi* (PP760158, Choi et al. 2024), *Tridacna crocea* (MK249738, Cai et al. 2019), *Tridacna derasa* (MG755811, Ma et al. 2018), *Tridacna gigas* (MT755623, Ma et al. 2020), and *Tridacna squamosa* (KP205428, Gan et al. 2016). The left phylogram presents the results of Bayesian analysis with node support values equal to 1, whereas the right tree reflects the maximum likelihood analysis with 100% bootstrap values. For BEAST2 reconstruction, the MCMC chain was run in four replicates for 10^7^ generations with sampling at every 10,000th step, relaxed log-normal clock, nucleotide substitution model GTR + I + 4G chosen as the best fitting by the bModelTest package in the BEAST2 software, and the Yule prior for the common tree. For the Iqtree2 method, default parameters, as well as the best-fitting GTR + F + I substitution model and ultrafast bootstrap approximation parameter set to 100,000 replicates were used. For the visualization of obtained relationships, the FigTree v1.4.4 (Rambaut 2018) graphical viewer was applied.

Analysis of the NGS dataset obtained from *C. glaucum* specimen collected in the Baltic Sea revealed a single putative mitochondrial contig, supporting the presence of a solitary mitogenome (GenBank accession number: PQ037489) with a total length of 14,953 base pairs (bp). The mitogenome of *C. glaucum* (Figure 2) encodes 13 protein-coding genes: *cox1-cox3*, *cytb*, *atp6*, and *atp8*, seven NADH subunit genes, two rRNA genes, and 22 tRNA genes: *trnT*, *trnC*, *trnI*, *trnL(tag)*, *trnP*, *trnA*, *trnS(tga)*, *trnD*, *trnN*, *trnY*, *trnE*, *trnF*, *trnH*, *trnG*, *trnV*, *trnM*, *trnW*, *trnL(taa)*, *trnK*, *trnS(tct)*, *trnQ*, and *trnR*. The 22 tRNA genes varied in length from 57 to 69 bp. The nucleotide composition in *C. glaucum* mtDNA is: 35.4% of T, 24% of G, 23.8% of A, and 16.8% of C with G–C content of 40.8%. The phylogenetic reconstructions (Figure 3) placed the reported Baltic Sea *C. glaucum* on one clade with *C. glaucum* (OZ205200) from England with 0.027 nucleotide *p*-distance for the whole mitogenome sequence, additionally, the genetic variation in the protein-coding region was estimated separately for synonymous and non-synonymous sites, resulting in  *π*_S_= 0.121 and *π*_a_ = 0.006, respectively.

## Discussion and conclusions

The reported complete mitochondrial genome of *C. glaucum* from the Baltic Sea exhibits a length comparable to that of *C. glaucum* (OZ205200) from England (14,950 bp) and *C. edule* (MF374632) (14,947 bp), which has a similar gene arrangement. The sequence divergence between the Baltic and English *C. glaucum* mitogenomes is predominantly associated with differences at synonymous sites, with extremely weak signal of non-synonymous substitutions. This pattern suggests the presence of natural intra-species polymorphism rather than adaptive divergence. No signs of heteroplasmy was detected in the gonadal tissue of male *C. glaucum*, contrasting with findings in other bivalve species where the NGS approach has revealed the coexistence of two highly divergent mitogenomes in mature male gonads (Śmietanka et al. 2018; Lubośny et al. 2020). These results support the hypothesis that mitochondrial DNA in *Cerastoderma* is transmitted exclusively through the maternal lineage (standard maternal inheritance – SMI model). The annotated mitogenome of *C. glaucum* includes 22 tRNA genes. In contrast, the mitogenome of *C. edule* (MF374632, unpublished) contains an additional tRNA gene, *trnM2*. However, none of the bioinformatic tools employed in this study identified *trnM2* as a functional tRNA gene, suggesting that the annotation of 23 tRNAs in *C. edule* may include a nonfunctional or pseudogenic artifact. Specifically, the ARWEN tool failed to recognize the trnM-like sequence as a valid tRNA, reinforcing the likelihood of its nonfunctional status. A similar annotation discrepancy has been observed in other Cardiidae species. For instance, *Acanthocardia tuberculata* (DQ632743) includes a manually predicted trnM2 gene (Dreyer and Steiner 2006), lacking computational validation. Notably, the anticodon of this putative trnM2 gene matches that of the canonical *trnM(cat)*, unlike the *trnM(tat)* anticodon found in the *trnM2* of *Mytilus* (Hoffman et al. 1992). The analytical pipeline applied in this study did not detect any spurious tRNA genes in DQ632743. Similarly, the mitogenome of *Keenocardium buelowi* includes only 22 tRNA genes, with a single trnM gene (Choi et al. 2024). The G + C content of the *C. glaucum* mitogenome is slightly lower (40.8%) than that of *C. edule*, which reaches 41.7%. These newly obtained mitogenomic data provide a valuable foundation for future studies focused on species identification, population genetics, and phylogeographic analyses within the genus *Cerastoderma*.

## Supplementary Material

Supplementary material for review.docx

## Data Availability

The genome sequence data are openly available in GenBank of NCBI at https://www.ncbi.nlm.nih.gov/ under the Accession No. PQ037489. The associated BioProject, SRA and Bio-Sample numbers are: PRJNA1134715, SRR29782653, and SAMN42435018, respectively.
