# Supplementary material for: The complete annotated mitochondrial genome of Cerastoderma glaucum (Bruguière, 1789) from the Baltic Sea
Source: Mitochondrial DNA B Resour. 2025 Nov 11;10(12):1164–8. doi: 10.1080/23802359.2025.2584950 (PMC12613299; doi:10.1080/23802359.2025.2584950)
Supplement: Supplementary material for review.docx [file TMDN_A_2584950_SM7548.docx]

**The complete annotated mitochondrial genome of *Cerastoderma glaucum* (Bruguière, 1789) from the Baltic Sea**

Beata Śmietanka

Department of Genetics and Marine Biotechnology, Institute of Oceanology Polish Academy of Sciences, Sopot 81-712, Poland

[bsmietanka@iopan.pl](mailto:bsmietanka@iopan.pl)


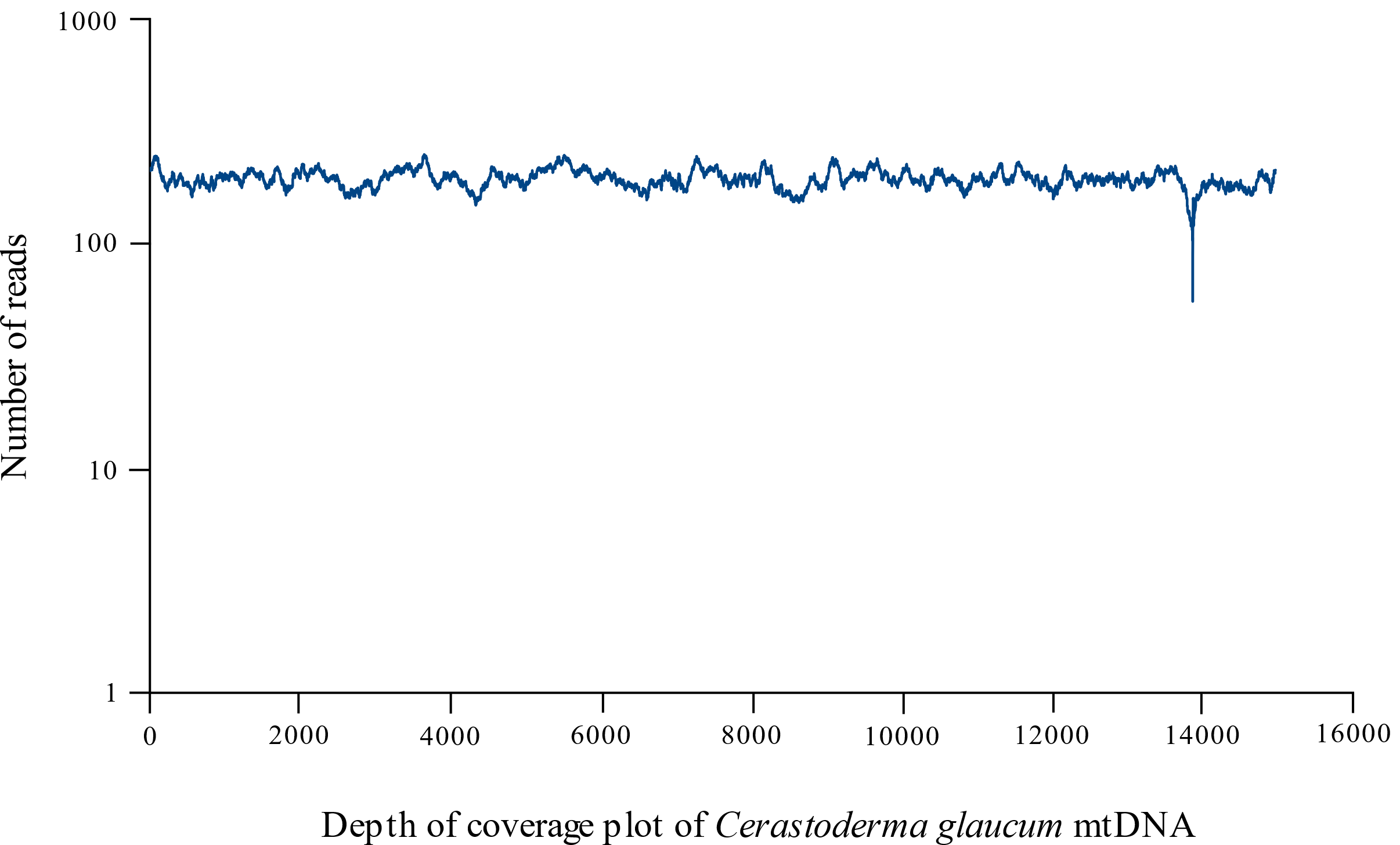


Figure S1. Read coverage plot of the *Cerastoderma glaucum* mtDNA from the Baltic Sea. It was obtained by mapping raw reads back at the final assembly, and counting the number of reads at each position of the mapping.
